# Safety, Tolerability, Pharmacokinetic and Pharmacodynamic Effects of the Muscarinic M 1 Positive Allosteric Modulator VU0467319 for Alzheimer’s disease: A Single Ascending-Dose Study in Healthy Participants

**DOI:** 10.21203/rs.3.rs-6271510/v1

**Published:** 2025-04-04

**Authors:** Alexander C. Conley, Alexandra P. Key, Jennifer U. Blackford, Jason K. Russell, Kimberly M. Albert, Xuewen Gong, Michael Bubser, Jerri M. Rook, P. Jeffrey Conn, Craig W. Lindsley, Carrie K. Jones, Paul A. Newhouse

## Abstract

The development of cholinergic neurotransmitter based cognitive enhancers for Alzheimer’s disease and other neuropsychiatric disorders have focused recently on allosteric modulation of specific muscarinic acetylcholine receptor (mAChR) subtypes to reduce dose-limiting side-effects that have been the hallmark of earlier orthosteric mAChR agonists. VU0467319 (VU319) is an investigational positive allosteric modulator of the M _1_ mAChR. A Phase 1 first-in-human study was conducted assessing safety and brain activity utilizing cognitive tasks and event-related potentials (ERPs) in single-ascending dose and food effect studies. VU319 was given orally to 52 healthy volunteers aged 18–55 years. The single ascending dose study tested 40 participants in five dose escalating cohorts (60, 120, 240, 400, 600 mg; 6 VU319/2 placebo per dose). The food effect study involved 12 participants, 10 VU319 (120 mg)/2 placebo. Exploratory cognitive and electrophysiological tasks were examined pre-dosing and at 5 hours post-dose. Tolerability was good with no observed dose limiting side effects throughout the full dose range tested. Drug exposure increased with dose in a less than dose-proportional manner with a half-life ranging from 36 to 43 hours. Absorption was increased with food. Exploratory cognitive/ERP testing showed evidence for drug-induced CNS activity on higher doses of VU319 compared to placebo. Single dose VU319 across five ascending cohorts appeared to have a favorable safety profile and a PK profile consistent with once daily dosing. Target engagement results suggest stimulation of the cholinergic system functioning in healthy adults following a single dose of VU319. These results provide a strong foundation for further studies of positive allosteric modulators of muscarinic M _1_ receptors for potential cognitive or behavioral benefits.

### Introduction

Alzheimer's disease (AD) is a chronic and irreversible neurodegenerative disease characterized by progressive deterioration of memory and other cognitive functions, impairments in normal daily living, and severe neuropsychiatric symptoms and behavioral disturbances (1, 2). Recent approvals of disease-modifying treatments (DMTs) have been revolutionary for the field, as anti-amyloid monoclonal antibodies have demonstrated the potential for limiting the progression of cognitive decline. Despite these advances, there are caveats with these new therapeutics, as they can have potentially serious adverse effects, related to the occurrence of amyloid-related imaging abnormalities (ARIA), which consist of edemas or microhemorrhages while cleaving amyloid from the blood vessels around the brain (3). The slowing of disease progression across an 18-month period for the three monoclonal antibodies as measured by Clinical Dementia Rating sum of box score was under 30%, with disease progression continuing (4). In addition, the use of monoclonal antibodies is logistically complex, requiring infusion clinics and regular neuroimaging for the detection of ARIA. In addition to DMTs, there is still a large unmet need for improved symptomatic therapies to improve cognitive and behavioral functioning in AD and other disorders that may benefit from cholinergic stimulation (e.g. schizophrenia).

The progression of cognitive decline in AD is typified by the deterioration of central cholinergic neurotransmission from the basal forebrain, which results in impairments of attention and memory (5–8). These deficits become present in the early stages of AD, prior to the widespread deterioration seen at later stages (1, 2, 9). Acetylcholinesterase inhibitors (AChEIs) are modestly helpful in these early stages of AD; however, their effectiveness is limited due to nonspecific upregulation of cholinergic transmission, narrow therapeutic index, and the progression of the disease (10–12). One alternative strategy has been to target the activation of the $M_1$ muscarinic acetylcholine receptor (mAChR) subtype. Of the five mAChR subtypes, the $M_1$ mAChR subtype is a promising target, as these receptors are highly expressed in the hippocampus, cortex and striatum, and are thought to be important for normal cognitive and affective function (13, 14). The impairment of M1 receptors has been shown to impact attention and memory performance in rodent animal models (15, 16). However, attempts to develop orthosteric agonists of the $M_1$ mAChRs have been limited by a lack of mAChR subtype selectivity due to the high conservation of the orthosteric binding site of Ach (17); resulting in dose-limiting adverse effects from excessive activation of peripherally expressed mAChRs, including salivation, lacrimation, urination, diaphoresis, gastrointestinal (GI) problems and emesis (SLUDGE) symptoms (18). While attempts to circumvent this problem have been developed with the combination of orthosteric agonists and peripheral muscarinic antagonists (e.g. Cobenfy, xanomeline/trospium combination; (19, 20)) other approaches to selective $M_1$ receptor activation are warranted.

In order to avoid the adverse side-effects associated with $M_1$ agonists, researchers have developed a novel strategy of selectively activating the $M_1$ mAChR through allosteric modulation (17, 21). Allosteric modulation of the mAChR enhances the impact of acetylcholine binding to the orthosteric site, which may reduce the likelihood of adverse off-target effects (22). Of the five mAChR subtypes, positive allosteric modulators (PAMs) targeting the $M_1$ mAChR subtype are promising targets, as these receptors are highly expressed in the hippocampus, cortex and striatum, and are thought to be important for normal cognitive and affective function (13, 14). The impairment of $M_1$ receptors has been shown to impact attention and memory performance in animal model (15, 16). Therefore, identification of candidate molecules that are highly selective for the $M_1$ mAChR has been seen as a way to focus the benefits of acetylcholine enhancement on cognitive processes.

Over the last decade, the focused efforts of the Warren Center for Neuroscience Drug Discovery (WCNDD) at Vanderbilt University have resulted in the discovery of the candidate molecule VU0467319 (VU319), a novel positive allosteric modulator (PAM) that selectively targets the $M_1$ mAChR subtype (23). $M_1$ PAMs in this class display robust $M_1$-mediated effects on hippocampal synaptic plasticity, excitatory drive to the PFC, and effects on both hippocampal and PFC-dependent cognitive functions in animal models that reflect cognitive domains impaired in AD or other neuropsychiatric disorders (14, 23–25). VU319 has shown to be effective at improving cognition in animal models, without producing SLUDGE effects (23).

Following analysis of its in vitro and in vivo pharmacologic and drug metabolism and pharmacokinetic (DMPK) profile, and completion of safety and toxicology in IND-enabling studies (23), we advanced this molecule to a first-in-human Phase 1 study. This study was a randomized, double-blind, placebo-controlled, ascending-dose, crossover study evaluating single oral doses of VU319 in healthy volunteers, with a food effect study to evaluate bioavailability in fed versus fasted conditions. We describe the safety, tolerability, and pharmacokinetic (PK) profiles of a single oral dose of VU319 in humans as well as evaluating acute effects on brain activity utilizing cognitive and electrophysiological performance markers.

## Methods

*Study Design and Participants*

This Phase 1 first-in-human safety and tolerability study of VU319 was conducted at the Vanderbilt University Medical Center Institute for Clinical and Translational Research (VICTR). The trial was registered on the Clinical Trial Registry (NCT03220295; 07/14/2017). All participants signed written informed consent prior to participation. This study was approved by the Duke University Institutional Review Board (IRB) and was performed in accordance with Good Clinical Practice guidelines and the Declaration of Helsinki.

Eligible participants were men and women without child bearing potential aged 18 through 55 years inclusive; with a body mass index of 18 through 32 kg/$m^2$; negative for Hepatitis B, Hepatitis C and HIV; a QTcF interval less than 450 ms in males and 470 ms in females; non-smokers or no nicotine use for at least 6 months; a negative urine screen for alcohol and drugs of abuse; and without clinically significant laboratory test results or electrocardiogram (ECG) abnormalities. In addition, participants agreed not to receive any vaccination within 21 days prior to admission and through Day 7 after final discharge; not to use nonprescription drugs, including vitamins, antacids, and herbal and dietary supplements within 7 days prior to admission and through 7 days after final discharge (except acetaminophen and ibuprofen at doses of ≤ 1 g/day); not to consume alcohol or caffeine for the 72 hours prior to admission and through 48 hours after discharge; not to eat grapefruit or drink grapefruit juice within 7 days prior to admission and through 24 hours after discharge; and not to eat or drink (except water) for 8 hours before and 4 hour after dosing for all single ascending dose cohort participants.

Exclusion criteria included an ongoing disease or disorder; clinically significant infection or inflammation at time of screening or admission; clinically significant abnormalities on physical/neurological exam at screening; acute gastrointestinal symptoms (e.g. nausea, vomiting, diarrhea) at time of screening or admission; a current, previous use of, or history of seeking advice from a physician or counselor for abuse or misuse of alcohol, non-medical drugs, medicinal drugs or other substance abuse, for example, solvents; use of medication (including OTC and oral contraceptive agents) within 14 days of admission that would affect the safety of the participant or any study assessment; a blood donation in the last 3 months; the use of any prohibited medication, or investigational medication within 3 months prior to the start of this study; and a history of severe allergies and/or adverse drug reactions.

Participants were screened up to 40 days prior to admission to the Vanderbilt Clinical Research Center (CRC). At admission, participants had baseline vital signs, clinical laboratory parameters and ECG were collected. The participants stayed overnight at the CRC, and in the morning performed the pre-dose cognitive and electrophysiological (ERP) batteries. Following completion of these batteries, pre-dose blood and urine for pharmacokinetic (PK) analysis was collected, as well as a second set of baseline vital signs. Participants then received an oral dose of VU319 or placebo with water. VU319 doses were assessed in five dose escalating cohorts of eight participants (60, 120, 240, 400 & 600 mg). The initial dose of 60 mg was chosen by allometric scaling of the highest observed adverse effect level (NOAEL) in animal species and reduced by a safety factor of 10. The increase in doses following the initial dose of 60 mg followed a modified Fibonacci sequence (26). In each cohort, six participants received oral VU319 while two participants received placebo. The randomization of participants to either placebo or active VU319 was done using a randomly permuted block procedure at a ratio of 1:3 for each of five dosages of VU319. Participants were required to remain in the CRC for 36 hours after dosing. Cognitive and ERP batteries were performed five hours post-dose. The decision to escalate to the next dose cohort was determined by review and approval from an independent safety monitoring committee, based on the safety and tolerability data from the preceding dose level.

The food effect study was completed after the first 5 ascending cohorts, participants were dosed once in a fasted state and once following a controlled high fat breakfast. Based on the PK results of the single ascending dose cohorts (see results), the 120 mg dosage group was chosen for the food effect study. This dose was chosen as it was a low-middle dosage in which the pharmacokinetic parameters could move up or down depending on the addition of food. The food effect study consisted of twelve participants, ten of whom received oral 120 mg VU319, while the other two received placebo. The participants received VU319 or placebo on two separate visits to the Vanderbilt CRC. These visits were separated by at least 14 days to allow for washout. On one visit, participants were dosed in a fasted state, while on the other visit, participants were dosed 30 minutes after eating a controlled high-fat breakfast. The order of the fed/fast conditions were randomized and balanced so that half the cohort were dosed fasting first, and the other half fed first. Post-dose procedures were shifted 30 minutes to allow for breakfast, but otherwise all study procedures remained the same as in the ascending dose cohorts.

*Safety and Tolerability*

Safety and tolerability were assessed throughout the study via treatment emergent adverse event (TEAE) monitoring, vital signs (blood pressure, pulse rate, respiration rate, oral temperature), physical examinations, 12-lead ECGs, and clinical laboratory parameters including complete blood count (CBC), coagulation, hematology, clinical chemistry assessments, and urinalysis. Safety laboratory tests were drawn at pre-dose and at 5, 24, 36 and 144-hours post-dose for 60-400 mg Cohorts; and at 24, 48, 72 and 144-hours post-dose for the 600 mg and Food Effect Cohort. ECG assessments were conducted at pre-dose and at 1, 2, 3, 5, 9, 24, 36 and 144-hours post-dose for 60-400 mg Cohorts; and at 2, 3, 5, 9, 24, 36, 72 and 144-hours post-dose for the 600 mg and Food Effect Cohort. All adverse events (AEs) were assessed by the investigator for severity and relationship to study medication or study procedures.

*Pharmacokinetic Sampling*

Blood samples (4 mL) were obtained by direct venipuncture into K2 EDTA tubes at 0.5 hours pre-dose and at 0.5, 1, 1.5, 2, 2.5, 3, 5, 7, 9, 12, 24, 26, 28, 36, 48 and 144-hours post-dose. Two blood-collection time points were moved from 0.5- and 1-hours post-dose to 72- and 96-hours post-dose for the 600 mg Cohort and the Food Effect Cohort due to the initial findings of a longer plasma half-life (>30 hours) of VU319 than originally estimated. Urine samples for PK quantification were collected at pre-dose and 2, 5, 12, 24, 36 and 144-hours post-dose. Urine volume was collected at 2, 5, 12, 24 and 36-hours post-dose to assess renal clearance.

Determination of plasma and urine concentrations of VU319 and metabolite VU0481424 (VU424) was carried out in compliance with Good Laboratory Practice using a validated method at Illinois Institute of Technology Research Institute (IITRI) laboratories. Pharmacokinetic endpoints included the following VU319 parameters for plasma: area under the plasma concentration-time curve ($AUC_{0-48}$); area under the plasma concentration-time curve extrapolated to infinity ($AUC_{0-\infty}$); percentage of the extrapolated area under the plasma concentration-time curve (%$AUC_{extrap}$); maximum observed plasma concentration ($C_{max}$); time (in hours) at which $C_{max}$ occurred ($T_{max}$); clearance ($C_{l/F}$); elimination half-life ($t_{1/2}$) and volume of distribution ($V_{d/F}$). Pharmacokinetic endpoints for VU319 for urine included the following parameters: renal clearance ($CL_u$); amount excreted in urine ($A_e$) and percent excreted in urine ($A_{e\%}$).

Exploratory Pharmacodynamics: Cognitive and Electrophysiological Measures

*Cognitive Battery: Attention Tasks*

The critical flicker fusion task (CFF; (27)) was used to test perceptual vigilance using the frequency of a flickering LED (between 12-50 Hz). The outcome variable for CFF is frequency (Hz) for ascending and descending trials. In the ascending trials, the participants pressed a button indicating when the light flickered to a speed where they appear to be continuously on or fused. In the descending trials, the participants pressed a button when the frequency of apparently fused lights is decreased such that lights begin to appear to be flashing.

The choice reaction time (CRT; (28)) task was used to measure psychomotor speed. Outcome variables on the CRT were the median total reaction time, which can be broken down into the recognition time (time from stimulus onset to initiation of movement), and the motor reaction time (time from initiation of movement to stimulus termination).

The Conner's Continuous Performance Task (CPT; (29)) tested sustained attention to a target. Participants saw a string of letters appearing one at a time on a computer screen. Participants were asked to press the spacebar whenever the target stimulus (any letter except for the X) appeared. Participants were asked to withhold responding when a X was presented, which is the non-target stimulus. A set of 15 outcome measures derived from the task report were recorded as key variables. These measures include Hit RT, Hit Standard Error, Commission, Omission and Perseveration errors, Sensitivity, Response style, and the Hit standard error across different interstimulus intervals.

A version of the spatial selective attention test (SSAT; (30)) was used to assess the ability of participants to disengage attention and shift to a new target. Participants were asked to press a button corresponding to the side of the screen on which a stimulus appeared. A central diamond was placed in between serving as a fixation point. Cues consisted of a 200-ms darkening of one side of the diamond depicting an arrowhead pointing to one of the peripheral boxes. The cue was followed by a target appearing for 100-ms in one of the boxes. To prevent temporal orienting, we used two randomly occurring cue–target intervals (400 and 700-ms). The targets were black diamonds that appeared in either the left or right boxes. In most trials, the cue validly predicted the target, however some cues were invalid, that is they pointed in the opposite direction of the target. On some trials, both sides of the diamond darkened, resulting in an ambiguous or neutral cue in which the participants were not able to prepare a specific hand to respond. On other trials, the cue did not show at all before the presentation of the target, giving participants no time to prepare a response. The task consisted of 112 trials, with 64 trials (57%) with valid cues, and 16 trials (14.3%) each for the invalid, neutral or no cues conditions. The outcome variables of the task are the reaction time effects related to the ability of participants to anticipate a target when there is an ambiguous cue compared to there being no cue at all (Alerting effect: no cue – neutral cue); and the ability of participants to shift their attention between congruent and incongruent stimuli (Reorienting effect: invalid cue – valid cue).

*Cognitive Battery: Memory Tasks*

A visually presented N-back sequential letter task (31,32) was used to assess working memory performance, wherein participants saw a string of consonants (except L, W, and Y), presented in upper case letters, one every 3 s. Four conditions were presented: 0-back, 1-back, 2-back, and 3-back. The 0-back control condition required participants to decide if the current letter matched a single target letter that was specified before the epoch began. In each of the 1-back, 2-back, and 3-back conditions, the task is to decide whether the letter currently presented matches the letter that has been presented 1, 2, or 3 back in the sequence. The 0-, 1-, 2-, and 3-back conditions were performed in two blocks of 27 trials each for a total of 216 trials. The blocks were presented in a counterbalanced order such that the same condition was not repeated two times in a row. The main outcome variable of the n-back task was sensitivity ($d'$) for each of the four conditions, calculated as: $d'$ = Z (Hit) – Z (False alarms).

The Selective Reminding Task (SRT; (33)) was used to test immediate and delayed episodic recall. Participants were read a list of 16 words and must immediately recall the list across 8 trials. Following each trial, participants were reminded of the words that they did not recall. Upon completing the immediate recall portion of the SRT and after a 20-minute delay, participants were asked to complete a single delayed recall trial. SRT total immediate recall was analyzed using the number of correctly recalled words across trials 1–8, total immediate recall consistency was analyzed using the number of words correctly recalled on two sequential trials across trials 1–8, SRT total immediate recall failure was analyzed using the number of words not recalled on two sequential trials across trials 1–8, and total delayed recall was analyzed using the number of words correctly recalled after a 20-min delay.

*Electrophysiological Recordings and Paradigms*

Following the cognitive battery, an electrophysiology recording was setup. Visual and auditory ERPs were recorded using a 128-channel Geodesic sensor net (EGI, Inc., Eugene, OR; (34,35)). The electrophysiological recording was sampled at 250 Hz with filters set at 0.1 Hz - 100 Hz. During data collection, all electrodes were referenced to vertex (Cz). The recording session lasted approximately 30 minutes.

Auditory and visual oddball tasks were performed to assess attention in auditory and visual modalities (36,37). In the auditory task, a pair of two pure tones (single formant) at 1000 and 1500 Hz were the stimuli. Tones were equated in duration (300 ms) and rise/decay times. Tones were presented at 75 dB SPL (measured at the ear) through a speaker positioned 1 meter in front of the participant. In the visual task, the stimuli were a pair of letters (X & O). All stimuli will be presented in random order for 500 ms in the center of a computer monitor positioned 1 meter in front of the participant. For both auditory and visual tasks, 200 trials were presented with the inter-stimulus interval varying randomly between 1000-1300 ms to prevent habituation to stimulus onset. The assignment of stimuli to standard and target conditions (70% & 30% of the trials respectively), was counterbalanced across participants. Each participant was asked to press a different button using their preferred hand upon presentation of the standard and the target stimulus. On average, auditory and visual tasks lasted 6-7 minutes each.

A passive incidental visual memory paradigm (120 trials, 8 min; (35)) was used to evaluate changes in basic memory processes. Participants were asked to view a slide show of novel color photographs depicting complex natural scenes (different sets of images were used at pre-dose vs. post-dose administration test sessions). A small subset ($n$ = 10) of the stimuli was presented repeatedly (× 5 each) throughout the session; the rest ($n$ = 50) were shown once. All stimuli were presented in random order for 1500 ms with a random interstimulus interval of 1300-1600 ms. To encourage attention to the stimulus sequence, 20 attention probes (a yellow smiley face) were presented throughout the test session and required a button press response. The task duration was approximately 7 minutes.

*Pharmacokinetic Analysis*

Single-dose PK parameters were derived from the plasma time concentrations and urinary concentrations. Pharmacokinetic parameters were summarized for each dose group using standard descriptive statistics. Summary statistics (mean, SD, min, max, coefficient of variation %) were calculated for each PK parameter. Plasma samples collected outside of the permitted windows (±5-minute for 0 to 48 hours, inclusive; ±3-hour for 72 to 144 hours, inclusive) were flagged, footnoted in listings of PK concentration data, and excluded from the summary statistics and mean graphs of concentration data. However, these values were used in the calculation of the PK parameters and were also included in the individual concentration/time listings and figures. In this study, no concentration values were excluded due to implausibility.

A noncompartmental PK method was used to analyze the concentration of VU319 and the primary metabolite, VU424. Individual and mean plasma and urine concentrations at each sampling timepoint were listed and descriptive summary statistics were calculated for each dose group/food condition. Time profile plots of each dose group/food condition, arithmetic and logarithmic means (±SE) at each time were generated. The dose proportionality of systemic exposure PK plasma parameters were evaluated using a power model. The PK parameters were log-transformed and evaluated using a linear mixed effects model that included dose as the fixed effect. Dose proportionality was analyzed by using the estimated slope of the model and 90% CIs that measured the degree of nonlinear proportionality.

For the evaluation of the food interaction, the effect of food on each of the systemic exposure PK parameters was evaluated using a mixed model with log-transformed PK-parameter values as the dependent variable. The model included sequence (fasted-fed or fed-fasted), period (1 or 2), and food condition (fasted or fed) as fixed effects, and participant nested within sequence as a random effect. Following antilog transformation of the results, the geometric mean ratios between food conditions and the corresponding 90% CIs were calculated with fasted condition as the reference.

Plasma and urine VU319 and VU424 concentration values that were below the lower limit of quantification (LLOQ) were displayed as "<LLOQ" in the data listings and were imputed as 0 for computing summary statistics and PK parameter calculations. Missing pre-dose PK concentrations were imputed as 0 in PK parameter calculations. Otherwise, missing data were not imputed. Actual post-dose sampling times were used in the calculation of plasma PK parameters and in the generation of individual concentration-time profiles. Nominal sampling times were used as a replacement for unknown or missing actual times and for the pre-dose values. Nominal sampling timepoints were used in the generation of mean plots and summary tables. For urine samples, nominal intervals were used. All PK analyses were performed using Statistical Analysis Software (SAS®; SAS Institute Inc., Cary, NC) version 9.4.

*Safety Analysis*

All AEs were coded using the Medical Dictionary for Regulatory Activities (MedDRA) version 22.0. All AEs were listed, and the emergence of TEAEs were categorized by body system affected and analyzed to determine whether they were related to treatment. Analysis of TEAEs for VU319 vs Placebo were completed using Fisher's Exact Test. Assessment of the grade of toxicity or relation to the study drug for each adverse event was determined by the Investigator. Changes in vital signs, physical examination, and laboratory tests were collected. Changes in vital signs from pre-dose to 48 hours post-dose were placed into 10-time bins and assessed using mixed-measures ANOVA with the time bins as the within-subjects factor and dose as the between-subjects factor. Analyses of safety data was completed by SAS version 9.4.

*Statistical Analyses for Exploratory Cognitive and ERP Data*

Due to the exploratory nature of these pharmacodynamic measures, we considered comparisons that resulted in *p*-values of 0.05-0.1 meaningful, if not statistically significant. As this study included small samples of participants who only received one dose of VU319, we placed a higher importance on the magnitude of the effect sizes than just statistical significance, as these indicated markers of potential examination by future studies.

Cognitive data: In this exploratory analysis, each outcome variable of the cognitive data was examined in a mixed model analysis of variance with one between-subjects factor of VU319 dose (0, 60, 120, 240, 400, 600 mg) and a within-subjects factor of testing session (pre-dose vs. post-dost administration). Post-hoc analysis compared each of the VU319 dose cohorts against the placebo group. Because the participants were healthy adults with normal cognitive function, we considered effects between no reductions in function to small improvement ($d = 0.0 – 0.20$) to be a positive result. For the food effect study, only the participants who received VU319 were examined due to the disproportionately small placebo group (n=2). A within-subjects ANOVA including food condition (fasted/fed) and testing session (pre-dose vs. post-dose administration) was performed on each outcome variable.

ERP processing and Analysis: Single-trial ERPs were derived by extracting a 100-ms pre-stimulus baseline and a 900 ms post-stimulus interval. The trials were screened for ocular and motor artifacts using automated procedures with standard threshold settings (NetStation 4.4, EGI, Inc) and verified using manual review. Data for electrodes characterized by consistent artifacts were replaced using the spherical spline interpolation algorithm (38,39). If more than 15 electrodes within a trial were noisy, the entire trial was discarded. Remaining artifact-free trials were averaged separately for each condition (standard vs. oddball; novel vs. repeated images), re-referenced to an average reference, and baseline corrected. For individual participant's data to be included in the analysis, each condition averages had to have a minimum of 10 artifact-free trials.

Statistical analysis focused on a priori selected electrode clusters corresponding to frontal, central, and parietal midline locations (Fz, Cz, Pz in 10-20 notation), confirmed using spatial principal components analysis. Mean amplitudes were calculated within

250-400 ms (frontal P3a), 300-500 ms (parietal P300), and 500-800 ms (parietal P600) temporal windows. These a-priori selected windows were confirmed using temporal principal components analysis.

Analyses for the auditory and visual oddball ERPs focused on the parietal (P300) and frontal (P3a) responses to standard and target stimuli (37). Differences in the amplitude, latency, and scalp topography of the responses were examined. Averages for standard and target stimuli were based on the same number of trials to guarantee comparable signal-to-noise ratios across conditions. For both oddball tasks, increased positive amplitude of the P300 waveforms for target compared to standard stimuli indicates more effective processing of task-relevant stimuli. For the incidental memory paradigm, differences in the mean amplitude of the parietal P300/P600 responses to repeated vs. novel presentations. The amplitude of the parietal P300/P600 waveform measures stimulus recognition (35). A larger positive amplitude for repeated compared to novel images indicates spontaneous formation of a memory trace.

Associations between VU319 plasma concentrations and with Cognitive and ERP data: Additional analyses examined the relationship between the plasma levels of VU319 as well as the main pharmacokinetic measurements ($C_{max}$, $T_{max}$, and $AUC_{0\text{-}last}$), to the outcome measures of cognitive and ERP performance. PK levels were selected at timepoints +5 following VU319 administration as the closest timepoints in relation to the post-dose cognitive and ERP testing. Comparisons were made between the PK and post-dose performance only. To test difference in electrophysiological performance, difference waveforms were computed for the contrasting conditions in each ERP task (Oddball: Target – Standard; Incidental Memory: Repeated – Novel). Pearson correlations were performed between PK at +5 hours and all outcome variables. The participants who received VU319 in the five cohorts of participants of the single ascending dose study were combined into a single group of 30 participants. The ten participants in the food effect Study who received VU319 were assessed separately for each food condition (Fasted and Fed). As these comparisons were exploratory, corrections for multiple comparisons were not performed.

## Results

### Demographics

40 participants (8 female, mean 32 ± 11.3 years) completed the single ascending dose study. Baseline characteristics are displayed in Table 1. For the food effect study, 12 participants (6 female, mean 41.1 ± 14.4 years) completed the study. Following admission to the CRC, no participant withdrew from the study, and all participants completed follow-up assessments.

[INSERT TABLE 1 HERE]

### Pharmacokinetics

Pharmacokinetic measurements for the single ascending dose cohorts are displayed in Table 2, and plasma concentrations are shown in Figure 1. No dose-limiting toxicity occurred at any dose cohort. VU319 was rapidly absorbed with early and sustained plasma concentrations for up to 48 hours post-dose, then declined gradually until the end of sample collection (144 hours). As seen in Figure 1, VU319 concentration across the first 48 hours showed a distinctive double-hump pattern, particularly in the higher two doses, with concentration decreasing after the 48-hour mark. This double-hump pattern may be evidence of enterohepatic recirculation (40), or formulation-related factors. The PK analysis revealed that after oral administration of VU319 at single doses ranging from 60 mg to 600 mg, median $T_{max}$ values ranged from 5.0 to 9.5 hours. Mean terminal elimination half-life values varied across the dose range in a dose-independent manner from 30.4 to 55.4 hours. Mean oral clearance values ranged from 1.73 to 3.42 L/h, and the volume of distribution (Vz/F) ranged from 71.8 to 219 L. Based on the linear power model, the $\beta_1$ and associated 90% CIs for the $C_{max}$, $AUC_{0\text{-}last}$, and $AUC_{0\text{-}\infty}$, it was shown that the systemic exposures to VU319 were less than dose-proportional over the studied dose range (60 to 600 mg). When examining the higher doses (240 to 600 mg), the systemic exposures to VU319 were approximately dose proportional.

The plasma pharmacokinetic values of the primary metabolite of VU319, VU0481424 (VU424), ranged in peak metabolism from 24 to 47.9 hours post-dose across the cohorts. $C_{max}$ and $AUC_{0\text{-}last}$ of VU424 increased across dose escalating cohorts reaching the highest values in the 600 mg cohort. VU424 was gradually formed, lagged behind VU319 in the circulation, and cleared slowly with measurable concentrations up to the end of sample collection (144 hours). Data for the plasma pharmacokinetic values for VU424 are found in Supplementary Table 1.

The pharmacokinetic measurements of VU319 and VU424 in urine are displayed in Supplementary Tables 2 and 3 respectively. The percentage of cumulative fraction of the dose excreted unchanged in urine over the entire collection interval (Total %fe) was <0.1% at all dose levels. The Max Rate was low, and Total Ae was low relative to doses administered. Correspondingly, low values of $CL_R$ and $CL_{ratio}$ were observed while the value of $CL_{NR}$ was relatively high at all dose levels. The rates and amounts of VU424 excreted generally increased with dose but were small. The total amounts of VU424 excreted in urine were negligible (Total Ae <1% of the dose) at all dose levels.

Plasma PK measurements for VU319 in the food effect study are displayed in Table 2, and plasma concentrations curves are shown in Figure 2. Data for the plasma concentrations on VU424 in the food effect study are shown in Supplementary Table 1. Urine concentrations for VU319 and VU424 in the food effect study are shown in Supplementary Tables 2 and 3 respectively. No dose-limiting toxicity occurred across the fed or fasted sessions. Under the fasted condition, VU319 was rapidly absorbed with early and sustained plasma concentrations for up to 48 hours post-dose, then declined gradually until the end of sample collection (144 hours). Under the fed condition, higher concentrations compared to the fasted condition were observed early but were not sustained and declined thereafter until the end of sample collection (144 hours). Figure 2 displays that the concentration of 120 mg VU319 under fed conditions reaches a peak concentration near to that of 400 mg under fasted conditions. The PK analyses showed that VU319 showed a median $T_{max}$ of 5.11- and 5.02-hours post-dose under fasted and fed conditions, respectively. $C_{max}$, $AUC_{0\text{-last}}$, and $AUC_{0\text{-}\infty}$ values after dosing under the fasted condition were lower than under the fed condition. Mean terminal elimination half-life values were comparable with mean $t_{½}$ of 37.4 hours and 36.9 hours under fasted and fed conditions, respectively. The inter-participant variability (CV%) for VU319 systemic exposure parameters ($C_{max}$, $AUC_{0\text{-last}}$, $AUC_{0\text{-}\infty}$) ranged from approximately 24% to 38% under fasted and fed conditions. Mean oral clearance values ranged from 2.01 to 2.35 L/h, higher in the fasted condition. The volume of distribution (Vz/F) was also higher in the fasted condition, and ranged from 85.3 to 94.4 L.

[INSERT TABLE 2 HERE]

**Safety Results**

The listing of TEAEs that occurred more than twice for all dose cohorts of the single ascending dose study and the food conditions in the food effect study are reported in Table 3. An expanded list of all the adverse events is presented in Supplementary Table 4.

There were 47 TEAEs reported across the 5 cohorts. Of the 40 participants that completed the single ascending dose study, 25 participants (62.5%) experienced one or more TEAE. Of these TEAEs, only 4 were rated as potentially related to treatment. No TEAE was rated as serious, and no deaths or discontinuations due to TEAEs were observed. The most common TEAE across all doses was headache, followed by dizziness and nausea. However, for all TEAEs, the Fisher's Exact Tests did not show a significant difference between VU319 or placebo (all $p > 0.1$). The analyses of the vitals measurements over the first 48 hours post-dose showed no significant dose-by-time interactions on any parameter. Analysis of the QT intervals of from the ECG measurements across the study showed no significant dose-by-time interactions or main effects of dose group.

There were 20 TEAEs reported across the 12 participants who completed the food effect study. The most common TEAE across all doses was headache, followed by muscle aches. Six of the TEAEs were reported by the two participants in the placebo group. For the 14 TEAEs reported in the VU319 group, four were reported during the Fed condition. Most of these TEAEs were rated as mild in severity, with one headache that was rated moderate in the placebo group, and a moderate increase in ALT and AST levels reported by one participant in the VU319 group. This elevation of liver enzymes was only present after discharge from the inpatient unit (72 hours post-dose) during a follow-up measurement of laboratory values and returned closer to normal by the final lab evaluation (144 hours post-dose), thus was determined not to be a TEAE. For all the TEAEs, the Fisher's Exact Tests did not show a significant difference between food or drug conditions (all $p > 0.1$). The analyses of the vitals measurements over the first 48 hours post-dose showed no significant dose-by-time interactions. Additionally, there were no dose-by-time interactions in ECG.

[INSERT TABLE 3 HERE]

**Exploratory Pharmacodynamic Results**

Cognitive Battery

Results of the outcome measures for each cognitive tasks at each time and dosage group in the single ascending dose study and the food effect study are listed in Supplementary Tables 5 and 6 respectively.

*Attention Tasks*

The results of the analysis of the CRT in the single ascending dose study showed no effect of dose, session, nor a dose by session interaction for the total score (all $p > 0.1$). When the total score was divided into the recognition and motor components, the same pattern was observed, with no impact of either dose or testing session on performance (all $p > 0.1$). The results of the CRT in the food effect study displayed a significant interaction between food condition and session for the recognition subcomponent ($F(1, 9) = 6.98$, $p = 0.027$, $d = 1.76$), this effect was driven by participants having a quicker recognition time post-dose compared to pre-dose during the fasted condition, and there being no change across sessions during the fed condition. There were no effects seen for the motor or total times.

For the CFF, the results of the single ascending dose study displayed a significant impact of the testing session (time) for the ascending trial ($F(1, 34) = 5.8$; $p = 0.02$; $d = 0.827$). All groups except for the 120 mg cohort, showed an increase of median ascending frequency detected (mean = 2.24 Hz) at the post-dose testing session. However, there was no effect of dose, nor a dose by time interaction (both $p > 0.3$). By contrast with the ascending trial, there was no significant effect of dose, time, or their interaction on the descending trial (all $F < 1$). For food effect study, there was a trend of time of testing observed for the ascending condition, driven by improved performance for post- vs pre-dose sessions ($F(1,9) = 4.2$, $p = 0.07$, $d = 1.37$).

Analysis of the CPT in the single ascending dose study showed an interaction of dose and time on hit response time (see Figure 3, $F(1, 34) = 2.34$, $p = 0.063$, $d = 1.19$), with participants responding faster following higher doses of VU319 compared to pre-dose performance or placebo. There was a significant effect of time for omission but not commission errors ($F(1, 34) = 6.8$, $p < 0.02$, $d = 0.91$), with more omission errors post-dose compared to the pre-dose session. This effect was similar across dosage groups. There was no impact of the time or dose on commission errors or hit RT variability. Post-hoc analysis of the Hit RT showed that there was a significant difference between the 600 mg dose of VU319 and placebo, with participants in the 600 mg dose cohort responding faster from pre-dose to post-dose than participants in the placebo cohort (mean difference: -28.12, $t(1, 14) = 2.87$, $p = 0.012$, $d = 1.48$). There were no significant effects of food or time found in the food effect study on the CPT.

The results of the analysis of the SSAT in the single ascending dose study showed a significant impact of the testing session for the reorienting effect ($F(1, 34) = 5.7$, $p = 0.02$, $d = 0.81$). This effect was driven by all groups having larger reorienting effect post-dose compared to the pre-dose session. However, there was no effect of dose, nor a dose by time interaction (both $p > 0.3$). There was no significant effect of dose, time or their interaction on the alerting effect (all $p > 0.2$). In the food effect study no effect of either food condition or time was observed on the SSAT.

*Memory Tasks*

In the single ascending dose study, there was a significant effect of time on the $d'$ score of the working memory N-back task for all N-back blocks except 2-back (0-,1- and 3-back $p < 0.02$), with $d'$ increased post-treatment compared to pre-dose performance. The $d'$ score did not show a significant effect of dose, nor dose by time in any of the four conditions (all $p > 0.2$) although participants performed more accurately for higher dose groups than placebo. In the 1-back task, participants performed more accurately following 600 mg doses compared to placebo, and in the 2-back task, participants performed more accurately following 400 mg doses compared to placebo. Both showed robust effect sizes (600 mg 1-back $d'$: $t(1, 14) = 1.672$, $p = 0.12$, $d = 0.86$; 400 mg 2-back $d'$: $t(1, 14) = 1.678$, $p = 0.12$, $d = 0.87$). For the food effect study, the analysis of the N-back task revealed a significant effect of time of testing on 1-back and 2-back sensitivity (1-back: $F(1, 9) = 8.98$, $p = 0.015$, $d = 2.0$; 2-back: $F(1, 9) = 14.43$, $p = 0.004$, $d = 2.55$). However, this effect was not related to food condition.

For episodic memory, the results of the analysis of the SRT data in the single ascending dose study did not show a significant effect of dose, nor dose by time in any of the four outcome measures (all $p > 0.2$). There was a significant effect of time for all four scores (all $p < 0.02$), however this effect showed that recall performance was reduced post-dose compared to pre-dose performance. There was no impact of food or time observed on SRT performance during the food effect study.

<u>Electrophysiological Results</u>

Mean amplitudes over the parietal cortex for each task and trial type at time point for all dose groups in the single ascending dose study and food effect study are displayed in Supplementary Tables 7 and 8 respectively. Due to excessive artifact in electrophysiological recordings, three participants were removed from the analysis of the visual oddball task (one each from the placebo, 120 mg and 240 mg cohorts), and one participant from the analysis of the incidental memory task (from the 120 mg cohort) in the single ascending dose study. In the food effect study, one participant was removed from the analysis of the auditory and visual oddball tasks.

The results of the auditory oddball task in the single ascending dose study showed that while there was little effect of VU319 at higher doses, following lower doses of VU319 (60 & 120 mg), participants elicited a greater positive parietal amplitude for target stimuli versus standard stimuli, and this difference between trial types were greater post-dose than at the pre-dose session (60 mg: $t(1, 5) = -3.23$, $p = 0.002$, $d = 1.32$; 120 mg: $t(1, 5) = -1.56$, $p = 0.18$, $d = 0.64$). In the food effect study, participants displayed a significantly larger P300 amplitude to targets compared to standards during the post-dose session of the fasted condition and fed conditions (Fasted: $t(1, 9) = -2.52$, $p = 0.033$, $d = 0.8$; Fed: $t(1, 9) = -2.68$, $p = 0.25$, $d = 0.85$).

During the single ascending dose study, the results of the analysis of the visual oddball task showed that while there was no effect of VU319 at lower doses, at the 400 mg dose of VU319, participants elicited a greater positive parietal amplitude for target stimuli versus standard stimuli in the post-dose session compared to the pre-dose session ($t(1, 4) = -2.87$, $p = 0.05$, $d = 1.28$). This effect was not found in the 600 mg dose cohort ($t < 1$). For the food effect study, there was no difference for the fasted condition. During the fed condition, participants displayed a larger P300 amplitudes for targets compared to standards in both sessions (Pre-dose: $t(1, 8) = 2.47$, $p = 0.039$, $d = 0.82$; Post-dose: $t(1, 8) = 2.18$, $p = 0.061$, $d = 0.73$).

The results of the incidental memory task in the single ascending dose study showed that while there was little effect of VU319 at lower doses, at the higher doses of VU319 (400 & 600 mg), participants elicited a greater positive parietal amplitude for repeated compared to novel stimuli. The P300/P600 amplitudes for these dose groups were larger than the pre-dose session or the placebo cohort. These effects showed large dose-dependent effects sizes (400 mg: $t(1, 5) = -2.03$, $p = 0.098$, $d = 0.83$; 600 mg: $t(1, 5) = -2.09$, $p = 0.09$, $d = 0.86$). The waveforms for the 400 mg and 600 mg cohorts for both sessions in comparison to the placebo cohort is shown in Figure 4. In the food effect study, participants did not show a difference between the novel and repeated images on either session during the fasted condition (both $p > 0.2$). During the fed condition, there was a significant difference between repeated and novel images, with repeated images eliciting a larger P300/P600 amplitude compared to novel images (see Figure 5; $t(1, 9) = 2.89$, $p = 0.018$, $d = 0.91$). The increased positive amplitudes for repeat vs novel images over the parietal cortex following VU319 administration was indicative of improved passive recognition of stimulus repetition for the participants in dose cohorts and food conditions with higher VU319 concentration.

<u>VU319 Plasma Level and Cognitive/ERP Outcome Variables Interactions</u>

In the single ascending dose study, the performance of participants on the CRT in the post-dose session was correlated with plasma VU319 concentration. Higher PK concentrations at +5 hours post-dose, and larger $C_{max}$ values were associated with faster total reaction time on the CRT ($C_{max}$: r = -0.41, $p = 0.026$; PK +5: $r = -0.382$, $p = 0.037$). The motor reaction time component of the task also showed a significant negative relationship with $C_{max}$ ($r = -0.41$, $p = 0.025$). There was a significant negative relationship between the pre-post difference in CPT Hit reaction time and both $C_{max}$ and $AUC_{0\text{-last}}$; with faster responding post-dose being observed in participants with higher $C_{max}$ and $AUC_{0\text{-last}}$ ($C_{max}$: $r = -0.5$, $p = 0.005$; AUC $_{0\text{-last}}$: r = -0.58, $p < 0.001$). In the food effect study, post-dose recognition speed on the CRT in the fed condition was negatively associated with both $C_{max}$ and PK

concentration at +5 hours; displaying that faster post-dose recognition was associated with higher plasma VU319 ($C_{max:}$ $r$ = -0.85, $p$ = 0.002; PK +5: $r$ = -0.9, $p$ < 0.001).

When examining the ERP tasks, it was observed that for incidental memory task, during the single ascending dose study, higher $T_{max}$ and AUC $_{0\text{-last}}$ values were associated with higher post-dose parietal P300/P600 waveforms for repeated but not novel images ($T_{max}$: $r$ = 0.38, $p$ = 0.037; AUC $_{0\text{-last}}$: $r$ = 0.39, $p$ = 0.035). In the food effect study, VU319 PK values at +5 hours post-dose were positively associated with the post-dose amplitudes of the difference waveform (Repeated – Novel; $r$ = 0.74, $p$ = 0.015). This correlation indicates that participants who had larger VU319 concentration at +5 hours post-dose also had a larger P300/P600 amplitudes to repeat stimuli compared to novel stimuli.

## Discussion

This article reports the first in human experience with VU319, a selective $M_1$ mAChR positive allosteric modulator. The results indicate that, after oral administration in healthy volunteers aged 18–55, single doses of VU319 from 60 to 600 mg was generally well tolerated. Dose-normalized exposures for the 60, 120, 240, and 400 mg dose groups compared to the 600 mg group suggested that the exposure at 600 mg relative to the 60 and 120 mg dose groups were less than dose-proportional, and those at 600 mg relative to the 240 and 400 mg dose groups were approximately dose-proportional. Absorption was somewhat prolonged, with median $T_{max}$ in the 5 to 9.5-hour range. VU319 had a moderate plasma half-life (mean t½ 30–55 hours) and a high volume of distribution of the terminal phase (76–238 L). This profile is consistent with once-daily dosing for evaluation of the safety and efficacy profile of VU319 for extended periods. The bioavailability of VU319 was affected by food, under fed conditions $C_{max}$ was 51% higher than under fasted conditions, while AUC was insignificantly higher. $T_{max}$ and t½ were similar under fasted and fed conditions. Comparison of VU319 under fasted and fed conditions showed that the concentration-time profiles were qualitatively similar. The fraction of VU319 excreted in urine was < 0.1% of the administered dose. The negligible amount excreted unchanged in urine indicates that VU319 is cleared primarily via metabolism with no contribution of renal clearance. VU424, the major metabolite of VU319, was gradually formed, lagged behind VU319 in the circulation, and cleared slowly with measurable concentrations up to the end of sample collection. Exposure ratios of parent to metabolite were similar at the 60 to 240 mg dose levels with parent exposures consistently higher than metabolite. The parent-to-metabolite exposure ratios were lower at the higher doses (400 and 600 mg).

VU319 was well tolerated after single oral doses of 60 to 600 mg. Most TEAEs were of mild severity, and no serious TEAEs or discontinuations occurred. None of the TEAEs were considered related to treatment, and no evidence of a dose-dependency for specific TEAEs was noted in either the single ascending dose cohorts or the food effect study. The most common TEAEs (dizziness, headache, and nausea) were similar across both VU319 and placebo groups. Headache and dizziness were the most common AEs in the study; however, there was no clear relationship between dose and these AEs or their severity. A number of these TEAEs may have been driven by the absence of food during the ascending dose cohorts. The results of the food effect study show that TEAEs reported during the fasted condition were higher than in the fed condition despite higher plasma levels during the fed condition. A maximum tolerated dose (MTD) was not achieved in the study and dose escalation ceased when exposure levels reached predefined limits based on pre-clinical species experience.

Modulation of central cholinergic systems through muscarinic receptors has been shown to be essential in cognitive performance (41). Cholinergic activity reflects the interaction between signal-driven and knowledge-based processes and mediates the enhancement of attention to external and internal stimuli (41). Specifically, cortical cholinergic integrity represents the degree to which internal knowledge disagrees with the sensory inputs, i.e., the uncertainty between contextual information and incoming sensory inputs (42, 43). The $M_1$ mAChR is highly expressed in cortical and limbic regions and so is ideally placed to modulate cognitive function (44–46). Selective activation of $M_1$ mAChR has been reported to improve several cognitive functions in preclinical species, including memory functions, novel object recognition and paired associates learning, and executive and attentional functions (25, 47–50). Recently published data with the selective $M_1$ PAM TAK-071 in participants with cognitive impairment in Parkinson's disease revealed an improvement in a composite cognitive measure, including tests of attention, executive function, and memory (51).

The results of the exploratory pharmacodynamic measures are supportive of functional $M_1$ receptor target engagement with VU319. Analysis showed improvements in cognitive and ERP performance measures after higher plasma exposure of VU319 compared to placebo. Participants on the highest dose (600 mg) of VU319 responded significantly faster to targets on the continuous performance test compared to participants on placebo (effect size $d = 1.2$). Additionally, on the incidental memory task, participants who received the higher two doses of VU319 compared to placebo exhibited larger P300/P600 amplitudes in response to repeated compared to novel images ($d > 0.8$), indicative of better memory following passive exposure to the stimulus. Further, examination of the relationship between plasma levels of VU319 and cognitive performance showed that the reaction times on two psychomotor speed measures decreased in relation to increasing plasma concentration of VU319 ($r > 0.38$). Interestingly, the ERP results suggests a threshold effect with change in ERP amplitudes only occurring at the 400 and 600 mg doses. This comports with the results from the $C_{max}$ data which shows a significant increase in plasma exposure beginning at 400 mg. Additional support comes from the results of the within-subjects food effect cohort in which a positive correlation was found between plasma level and P300/P600 ERP increased amplitudes of difference waveform (repeat – novel) for participants during the fed condition which resulted in higher VU319 exposure.

The results of the visual oddball task displayed increased P300 amplitudes for target vs standard stimuli for the 400 mg dose of the single ascending dose study. The posterior P300 is related to the processing of task salient stimuli and evaluation for decision making, memory encoding, and updating working memory (37, 52). The waveform is generated in the temporal and parietal cortices (areas with high cholinergic activity) and reflects cognitive processes supported by cholinergic function (53), and is reduced in AD compared to healthy controls (54). Previous research has shown that cholinergic agents affect P300 amplitudes in both healthy adults and AD patients (52); with agonists like nicotine increasing amplitudes (55, 56) and antagonists like scopolamine reducing amplitudes (57). The results from the present study revealed that in higher concentrations, VU319 improved task salient stimulus processing on the visual oddball, which is consistent with this previous research and suggests that $M_1$ activation enhanced attention and arousal processes.

Other cognitive tests including the CRT and the CPT tasks showed that increasing VU319 concentration was significantly associated with the improvement (decrease) in reaction time for both tasks in both the ascending dose cohorts and the food effect cohort. Importantly, at no dose were adverse effects seen on cognitive performance which suggests that additional benefit from higher plasma levels than achieved in this study might be seen, at least acutely. Considering that post-dose testing occurred in the afternoon when cognitive performance often declines compared to early morning, the maintenance or improvement of performance is notable. The specificity of VU319 (lack of effects at other neurotransmitter receptors, lack of direct orthosteric agonism of $M_1$ receptors) suggests that the enhancement of ERP amplitude and improvements in reaction time post treatment likely reflects functional $M_1$ target engagement via the proposed allosteric mechanism. Dose relatedness of these effects adds additional confidence as these effects were only seen at higher doses while fasting or after higher plasma levels following lower dose administration with food. The relationship between VU319 exposure and electrophysiological performance on the incidental memory task is revealed in the positive associations between increased repeat vs novel amplitudes and the $T_{max}$ and $AUC_{0-last}$ values (the time of the post-dose testing corresponds to the $T_{max}$ of the 400 mg and 600 mg cohorts) as well as during the Food Effect cohort.

This study does not permit disentangling potential effects of VU319 on memory versus attention or both. Enhanced ERP amplitude in the passive memory task and the enhancement of psychomotor speed that were seen at higher doses may be explained by improvements in attentional circuits. This is consistent with the proposal by Sarter (41) and others (5) that a principal role of the cortical cholinergic system is to mediate attentional processes and that this system will be involved when attentional processes are needed because a task is difficult or relevant and irrelevant information is difficult to discriminate.

## Conclusion

Overall, VU319 was well-tolerated in healthy participants at single oral doses up to 600 mg. There were no deaths, serious TEAEs, or TEAEs leading to discontinuation in this study. Headache and dizziness were the most common AEs in the study; however, there was no clear relationship between dose and these AEs or their severity. No dose-limiting effects or apparently dose-dependent AEs were observed. VU319 exposure parameters ($C_{max}$, $AUC_{0-last}$, and $AUC_0$ ∞) increased in a less than doseproportional manner over the studied dose range (60 to 600 mg), but approximately proportional to dose in the higher dose groups (from 240 to 600 mg). The median $T_{max}$ ranged between approximately 5- and 10-hours post-dose, and mean T½ ranged from approximately 30 to 55 hours. Following a 120 mg dose of VU319 under fed conditions, $C_{max}$ was 51% higher than under fasted conditions, while area under the plasma concentration-time curve (AUC) was insignificantly higher.

While this study was not designed to examine pharmacodynamic effects of clinical relevance in neurodegenerative or neurocognitive disorders, these exploratory biomarkers were included to provide early evidence of potential functional target engagement of muscarinic cholinergic receptors. The results of the analyses of these exploratory results of VU319 showed evidence of functional engagement of VU319 with brain in both cognitive and electrophysiological tasks. The results of the present study suggest that at higher doses of VU319, cognitive performance and ERP morphology were enhanced compared to pre-dose performance or following placebo and in no cases were worse, despite post-dose testing occurring in the middle of the afternoon. In the cognitive battery, single doses of VU319 at 600 mg were able to improve CPT hit response time, while both 400 and 600 mg cohorts displayed improved accuracy on the N-back task. Electrophysiological evidence for both 400 and 600 mg cohorts indicated improved late incidental memory for repeated compared to novel images. The results of the exploratory pharmacodynamic measures are supportive of functional $M_1$ receptor target engagement with single dose administration of VU319 and suggest that this approach to enhancing muscarinic cholinergic $M_1$ receptor function may have potential for cognitive enhancement in longer term studies in patients with cognitive impairment secondary to Alzheimer's disease and related disorders.

## Abbreviations

$\%AUC_{ext}$: percentage of area under the curve extrapolated from time $T_{last}$ to infinity; AD: Alzheimer's Disease; AE: adverse event; Ae: amount excreted in urine; Ae%: percent excreted in urine; ALT: alanine aminotransferase; ARIA: amyloid-related imaging abnormality; AST: aspartate aminotransferase; $AUC_{0\text{-last}}$: area under the plasma concentration-time curves from time 0 to the time of the last detectable concentration; $AUC_{0\text{-last}}$/Dose: $AUC_{0\text{-last}}$ normalized to dose; $AUC_{0\text{-}\infty}$: area under the plasma concentration-time curve from time 0 to infinity; $AUC_{0\text{-}\infty}$/Dose: $AUC_{0\text{-}\infty}$ normalized to dose; BMI: body-mass index; CBC: complete blood count; CFF: Critical Flicker Fusion Task; CI: confidence interval; CL/F=apparent systemic clearance following nonintravenous (e.g., oral) administration; CLu: renal clearance; $C_{max}$: maximum observed plasma concentration; $C_{max}$/Dose=$C_{max}$ normalized to dose; CNS: central nervous system; CPT: Conners' Continuous Performance Task-II; CRC: clinical research center; CRT: Choice Reaction Time Task; DMT: disease-modifying therapy; EDTA: ethylenediaminetetraacetic acid; ECG: electrocardiogram; ERP: Event-related potential; h: hour Hz: frequency in Hertz; L: liter; LLOQ: lower limit of quantification; m: meter; mAChR: muscarinic acetylcholinergic receptor; MedDRA: Medical Dictionary for Regulatory Activities; mg: milligram; min: minutes; ms: millisecond; MTD: maximum tolerated dose; N: number of participants; NOAEL: no observed adverse event level; OTC: over-the-counter; PAM: positive allosteric modulator; PK: pharmacokinetic; QTcF: QT corrected for heart rate by Fridericia's cube root formula; RT: reaction time; SD: standard deviation; SE: standard error of the mean; SRT: Selective Reminding Task; SSAT: Selective Spatial Attention Task; $t_{½}$: apparent terminal elimination half-life; TEAE: treatment emergent adverse event; $T_{max}$: time to maximum plasma concentration; $T_{last}$: time of last detectable concentration; $V_z$/F: apparent volume of distribution following nonintravenous (e.g., oral) administration; μV: microvolt.

## Declarations

#### Acknowledgements

The authors thank the Vanderbilt Coordinating Center for their assistance in running the study, and all participants who took part in this study. Study data were collected and managed using REDCap electronic data capture tools hosted at Vanderbilt University Medical Center (58,59).

#### Funding

This work was supported by an Alzheimer's Association Part the Cloud grant PCTR-16-383171, Alzheimer's Drug Discovery Foundation grant 21040501.03 and a Clinical and Translational Science Award 2UL1TR000445 for the Vanderbilt Institute for Clinical and Translational Research.

#### Author Contributions

PAN was the principal investigator and grant recipient, APK and JUB were the sub-investigators, and XG was the study coordinator at the site. ACC was the study scientist, and was involved in the collection, analysis and interpretation of the data. JKR was involved in data collection and in the interpretation of the data. KMA was involved in the interpretation of the data. MB, JMR, PJC, CWL, and CKJ were involved in the preclinical studies. ACC and PAN wrote the manuscript; APK, JUB, JKR, KAM, MB, JMR, PJC, CWL and CKJ were involved in the review and editing of the manuscript.

#### Competing Interests

The authors have no competing interests to declare.

#### Data availability

The datasets analyzed during the current study are available from the corresponding author upon reasonable request.

#### Ethics approval and consent to participate

This study was approved by the Duke University Institutional Review Board and was performed in accordance with Good Clinical Practice guidelines and the Declaration of Helsinki. All participants signed written informed consent documents before participating in the study.

## Tables

Table 1. Demographic Information

| VU319 Study | | | | | | | | | |
|---|---|---|---|---|---|---|---|---|---|
| | Single Ascending Dose Study Cohorts | | | | | | Food Effect Study: 120 mg | | |
| Demographic Parameter | Placebo (N=10) | 60 mg (N=6) | 120 mg (N=6) | 240 mg (N=6) | 400 mg (N=6) | 600 mg (N=6) | Placebo (N=2) | Fasted-Fed Sequence (N=5) | Fed-Fasted Sequence (N=5) |
| Sex; Male : Female | 8 : 2 | 5 : 1 | 5 : 1 | 5 : 1 | 5 : 1 | 4 : 2 | 0 : 2 | 4 : 1 | 2 : 3 |
| Age at Screening (years) | | | | | | | | | |
| Mean (SD) | 30.1 (9.75) | 33.0 (11.0) | 37.3 (14.2) | 29.8 (9.9) | 33.0 (11.15) | 33.3 (6.2) | 53.0 (2.83) | 39.8 (6. 32) | 38.0 (17.2) |
| Range | 20-53 | 23-51 | 20-51 | 19-48 | 21-52 | 20-54 | 51-53 | 19-53 | 18-54 |
| Height (cm) | | | | | | | | | |
| Mean (SD) | 171.04 (4.4) | 178.57 (7.9) | 171.05 (11.02) | 179.67 (10.93) | 176.12 (9.71) | 170.45 (12.86) | 163.0 (7.07) | 174.10 (4.64) | 166.60 (10.36) |
| Range | 162.5-176.0 | 166.0-186.0 | 152.0-183.0 | 162.0-191.0 | 164.0-190.0 | 155.0-187.0 | 158.0-168.0 | 167.0-180.0 | 152.0-181.0 |
| Weight (kg) | | | | | | | | | |
| Mean (SD) | 73.8 (11.24) | 72.8 (14.18) | 87.55 (10.34) | 79.88 (3.96) | 84.33 (19.28) | 68.73 (13.13) | 75.65 (17.47) | 80.46 (5.23) | 66.76 (12.88) |
| Range | 60.2-92.6 | 55.8-93.3 | 69.7-95.3 | 74.1-84.6 | 62.7-111.6 | 50.8-82.6 | 63.3-88.0 | 73.0-86.3 | 49.9-83.7 |
| BMI (kg/$m^2$) | | | | | | | | | |
| Mean (SD) | 25.24 (3.76) | 22.69 (3.16) | 29.89 (1.88) | 25.0 (3.5) | 26.88 (3.63) | 23.53 (2.97) | 28.27 (4.12) | 26.66 (3.08) | 23.97 (3.72) |
| Range | 20.1-31.3 | 18.6-27.9 | 28.2-33.4 | 21.7-29.9 | 22.0-30.9 | 20.0-28.7 | 25.36-31.2 | 22.5-30.9 | 20.5-29.3 |

Notes. Range = Minimum - Maximum; N = number of participants in each group; n=number of participants meeting the criterion; SD = standard deviation; BMI = body mass index.

Table 2. Plasma Pharmacokinetic Parameters of VU319

| | Single Ascending Dose Cohorts | | | | | | | | | | Food Effect Study | | | |
|---|---|---|---|---|---|---|---|---|---|---|---|---|---|---|
| VU319 Dose | 60 mg | | 120 mg | | 240 mg | | 400 mg | | 600 mg | | 120 mg Fasted Condition | | 120 mg Fed Condition | |
| PK Parameter | Mean (SD) | N | Mean (SD) | N | Mean (SD) | N | Mean (SD) | N | Mean (SD) | N | Mean (SD) | N | Mean (SD) | N |
| $C_{max}$ (μg/mL) | 0.725 (0.209) | 6 | 0.884 (0.312) | 6 | 1.18 (0.425) | 6 | 1.72 (0.447) | 6 | 2.81 (0.64) | 6 | 0.985 (0.38) | 10 | 1.42 (0.344) | 10 |
| $T_{max}$ (h)[a] | 7.00 (2.50, 9.00) | 6 | 5.00 (2.00, 24.0) | 6 | 9.50 (3.00, 24.0) | 6 | 5.98 (2.02, 48.2) | 6 | 5.01 (2.00, 48.0) | 6 | 5.11 (2.50, 48.1) | 10 | 5.02 (2.50, 8.97) | 10 |
| $AUC_{0\text{-}last}$ (μg•h/mL) | 36.8 (10.9) | 6 | 60.1 (13.7) | 6 | 63.8 (18.4) | 6 | 132 (47.4) | 6 | 207 (47.1) | 6 | 56.8 (18.0) | 10 | 62.5 (15.6) | 10 |
| $AUC_{0\text{-}\infty}$ (μg•h/mL) | 39.2 (13.3) | 6 | 59.0 (9.07) | 5 | 78.3 (15.2) | 4 | 124 (32.0) | 4 | 221 (54.6) | 6 | 60.7 (23.3) | 8 | 66.6 (19.3) | 9 |
| $\%AUC_{ext}$ (%) | 4.81 (6.18) | 6 | 6.30 (3.55) | 5 | 5.48 (6.31) | 4 | 11.2 (4.73) | 4 | 5.73 (2.41) | 6 | 5.86 (4.51) | 8 | 5.79 (4.31) | 9 |
| CL/F (L/h) | 1.73 (0.71) | 6 | 2.07 (0.28) | 5 | 3.15 (0.58) | 4 | 3.42 (0.96) | 4 | 2.87 (0.77) | 6 | 2.35 (1.15) | 8 | 2.01 (0.85) | 9 |
| $V_z/F$ (L) | 71.8 (20.2) | 6 | 107 (6.4) | 5 | 143 (52.7) | 4 | 219 (21.6) | 4 | 123 (23.8) | 6 | 94.4 (27.8) | 8 | 85.3 (16.4) | 9 |
| $t_{½}$ (h) | 31.5 (13.3) | 6 | 55.4 (46.8) | 6 | 35.0 (14.5) | 5 | 46.2 (9.54) | 4 | 30.4 (4.37) | 6 | 37.4 (15.9) | 10 | 36.9 (17.0) | 10 |
| $C_{max}$/Dose (ng/mL/mg) | 12.1 (3.49) | 6 | 7.36 (2.60) | 6 | 4.91 (1.77) | 6 | 4.30 (1.12) | 6 | 4.68 (1.07) | 6 | 8.21 (3.144) | 10 | 11.84 (2.87) | 10 |
| $AUC_{0\text{-}last}$/Dose (ng•h/mL/mg) | 613 (182) | 6 | 501 (114) | 6 | 266 (76.6) | 6 | 330 (119) | 6 | 346 (78.5) | 6 | 473.6 (150) | 10 | 520.6 (130) | 10 |
| $AUC_{0\text{-}\infty}$/Dose (ng•h/mL/mg) | 653 (221) | 6 | 491 (75.6) | 6 | 326 (63.3) | 6 | 309 (79.9) | 6 | 368 (91.0) | 6 | 506 (194.5) | 8 | 555.33 (160.44) | 9 |

Notes. $\%AUC_{ext}$=percentage of area under the curve extrapolated from time $T_{last}$ to infinity; $AUC_{0\text{-}last}$=area under the plasma concentration-time curves from time 0 to the time of the last detectable concentration; $AUC_{0\text{-}last}$/Dose=$AUC_{0\text{-}last}$ normalized to dose; $AUC_{0\text{-}\infty}$=area under the plasma concentration-time curve from time 0 to infinity; $AUC_{0\text{-}\infty}$/Dose=$AUC_{0\text{-}\infty}$ normalized to dose; CL/F=apparent systemic clearance following nonintravenous (e.g., oral) administration; $C_{max}$=maximum observed plasma concentration; $C_{max}$/Dose=$C_{max}$ normalized to dose; N=number of participants; PK=pharmacokinetic; SD=standard deviation; $t_{½}$=apparent terminal elimination half-life; $T_{max}$=time to maximum plasma concentration; $T_{last}$=time of last detectable concentration; $V_z/F$=apparent volume of distribution following nonintravenous (e.g., oral) administration. [a]$T_{max}$ values are presented as median (minimum, maximum).

Table 3. Adverse Events Across Single Ascending Dose Cohorts and the Food Effect Study

| Participants, N (%) | | | | | | | | | |
|---|---|---|---|---|---|---|---|---|---|
| | **Single Ascending Dose Study** | | | | | | **Food Effect Study** | | |
| **MedDRA System Organ Class**<br><br>**Preferred Term** | **Placebo (N=10)** | **60 mg (N=6)** | **120 mg (N=6)** | **240 mg (N=6)** | **400 mg (N=6)** | **600 mg (N=6)** | **Placebo (N=4)** | **120 mg Fasted Condition (N=10)** | **120 mg Fed Condition (N=10)** |
| **Participants with any TEAE** | 6 (60.0) | 4 (66.7) | 3 (50.0) | 4 (66.7) | 4 (66.7) | 4 (66.7) | 3 (75.0) | 7 (70.0) | 2 (20.0) |
| **Nausea** | 2 (20.0) | 1 (16.7) | 1 (16.7) | | | | | | |
| **Myalgia** | | | | | 1 (16.7) | | | 1 (10) | |
| **Headache** | 2 (20.0) | 3 (50.0) | 2 (33.3) | 1 (16.7) | 3 (50.0) | 3 (50.0) | 3 (75.0) | 4 (40.0) | 1 (10.0) |
| **Dizziness** | | | | 3 (50.0) | | | | | |
| **Rhinorrhea** | 2 (20.0) | | | | | | | | |

Abbreviations: MedDRA=Medical Dictionary for Regulatory Activities; N=number of participants in each group; n=number of participants meeting the criterion; TEAE=treatment-emergent adverse event. Notes: Listed TEAEs are for those occurring in ≥ 2 participants overall, a more detailed list of TEAEs is found in the Supplementary Materials. The number of participants (N) for the given dose group is used as the denominator for calculating percentages for the participant counts. A TEAE is an adverse event with onset date on or after the first study dose date and no later than last study dose date + 30 days. A participant may have more than one TEAE per category. A participant is counted at most once per category. For events, all occurrences of TEAEs are counted per category.

### Figures

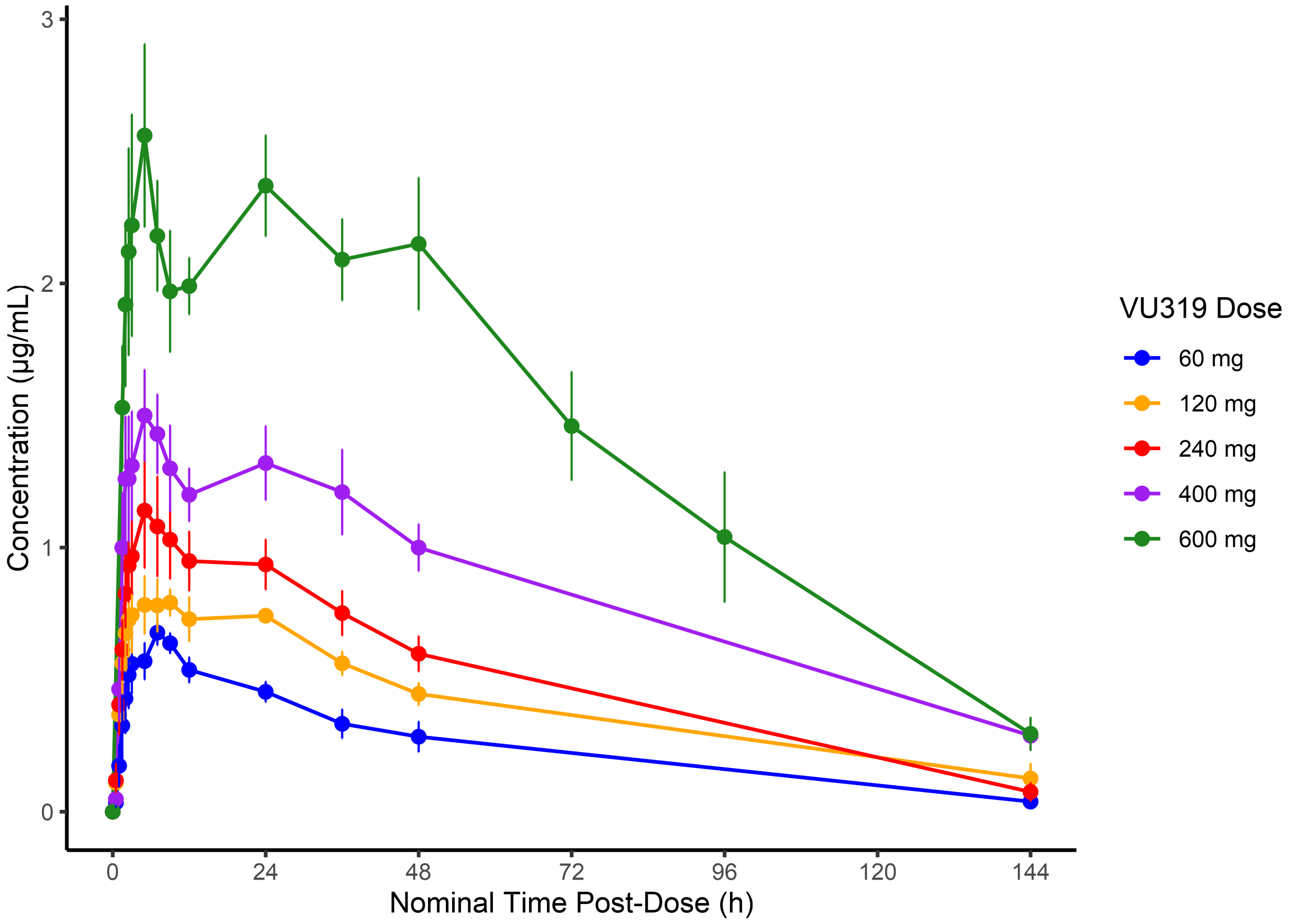

**Figure 1**

Mean (SE) plasma concentrations versus time profiles for the single ascending dose cohorts of VU319.

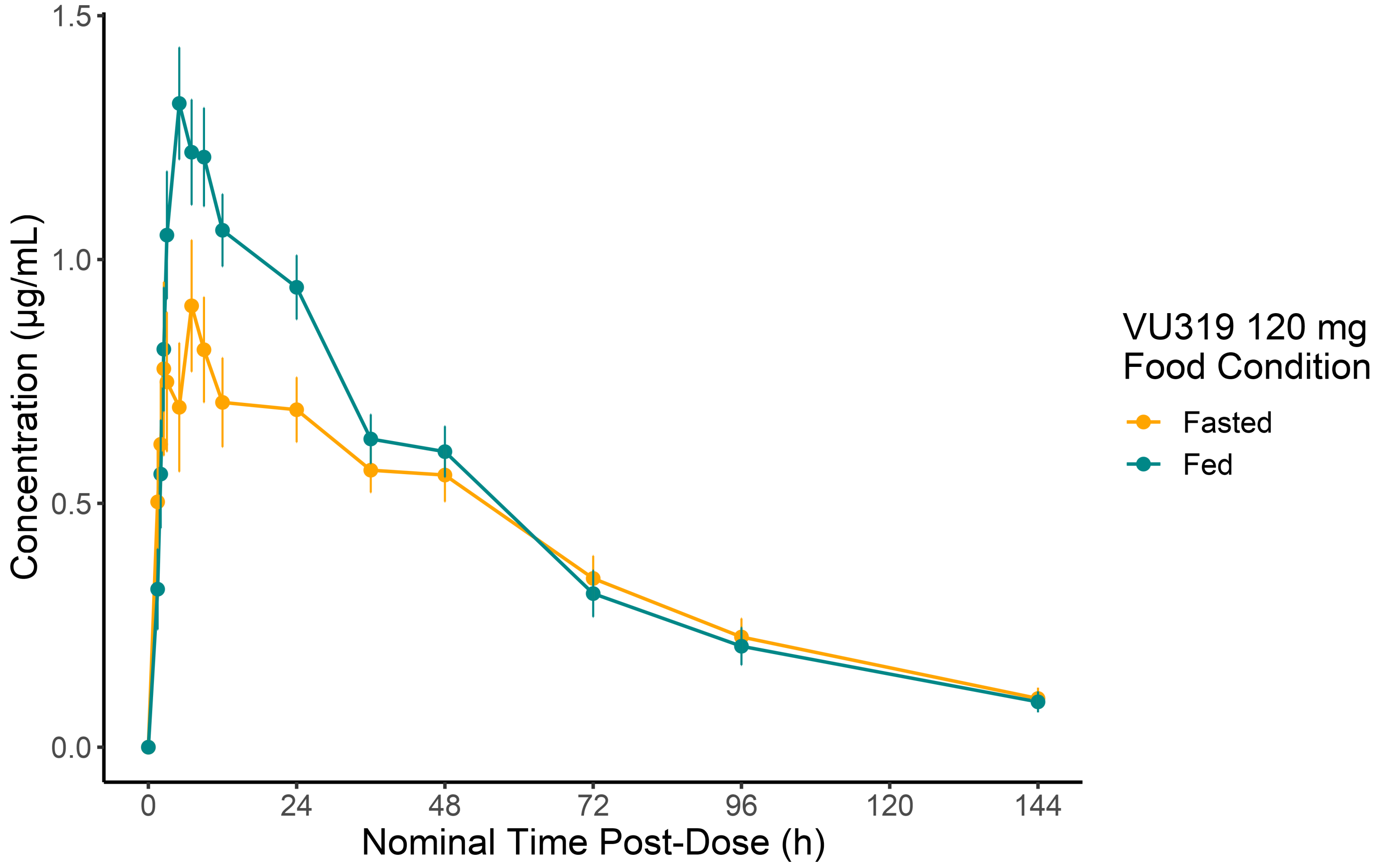

**Figure 2**

Mean (SE) plasma concentrations versus time profiles for the food effect study of VU319.

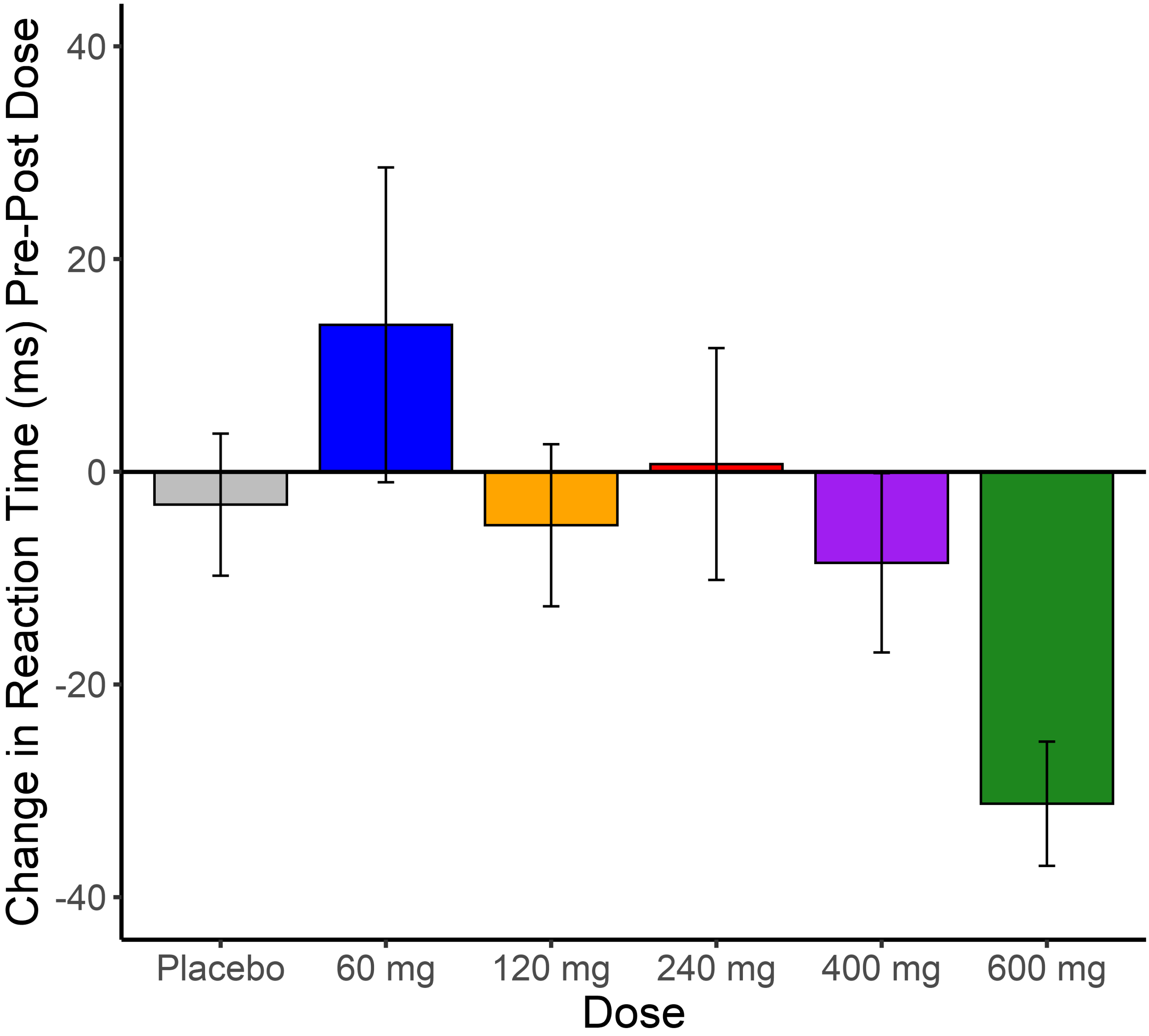

**Figure 3**

CPT Hit reaction time represented as the difference in response time from pre-drug (Post-Dose – Pre-Dose).

Legend. Negative scores represent faster response times post-dose, and positive scores represent slower response times post-dose. Error bars are the standard error of the difference scores.

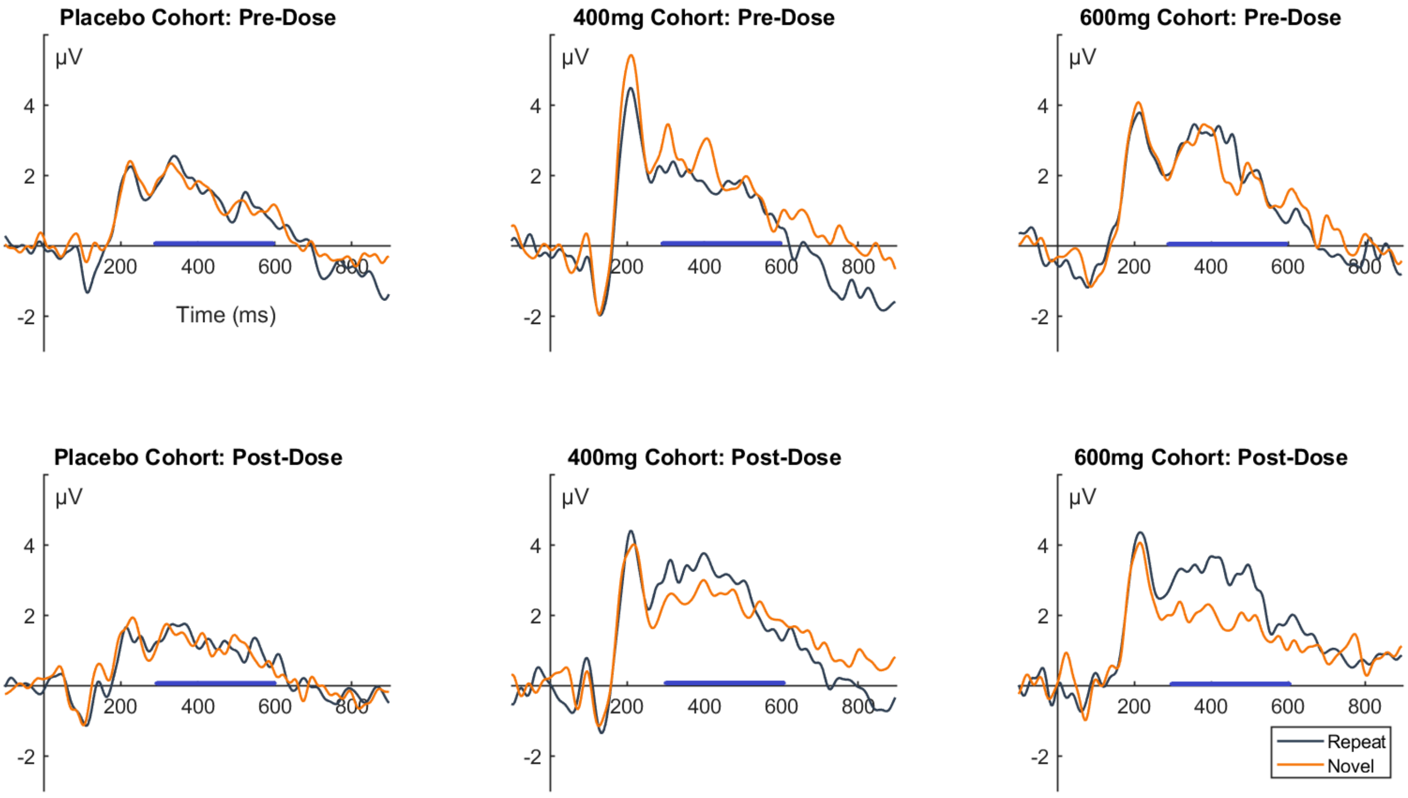

**Figure 4**

Event-related potential (ERP) waveforms of incidental memory task performance of the Single Ascending Dose study.

Legend. Listed are the ERP waveforms for the Placebo, 400 mg and 600 mg cohorts of the Single Ascending Dose study. The waveforms are all from the parietal cluster, and separate plots are displayed for the pre-dose and post-dose sessions. The mean amplitude windows for the P300/P600 waveform is denoted by the shaded part of the x-axis in each plot.

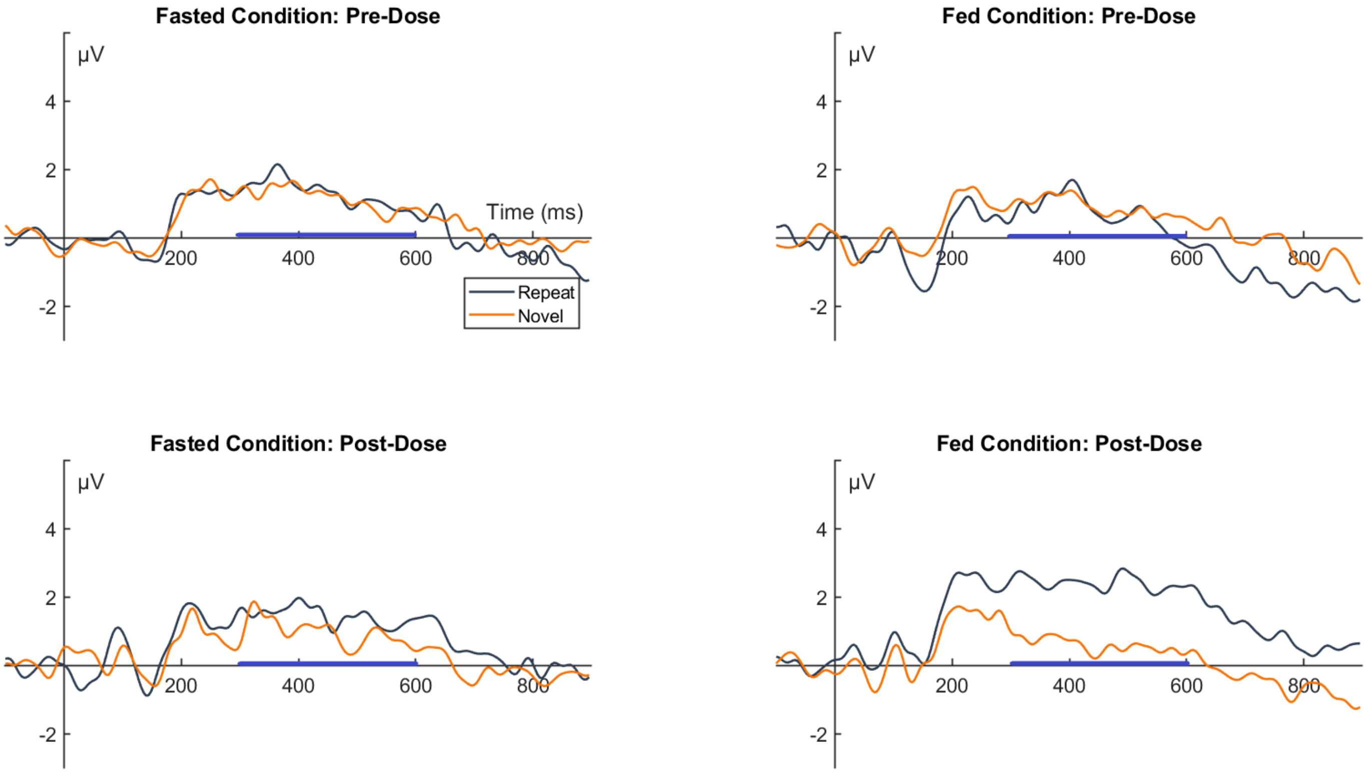

**Figure 5**

Event-related potential (ERP) waveforms of incidental memory task performance for the Food Effect study.

Legend. Displayed are the waveforms for the Fasted and Fed conditions of the food effect study. The waveforms are all from the parietal cluster, and separate plots are displayed for the pre-dose and post-dose sessions. The mean amplitude windows for the P300/P600 waveform is denoted by the shaded part of the x-axis in each plot.

### Supplementary Files

This is a list of supplementary files associated with this preprint. Click to download.

- ConleyetalSupplementaryMaterial.docx
